# Topsensterols A–C, Cytotoxic Polyhydroxylated Sterol Derivatives from a Marine Sponge *Topsentia* sp.

**DOI:** 10.3390/md14080146

**Published:** 2016-08-01

**Authors:** Min Chen, Xu-Dong Wu, Qing Zhao, Chang-Yun Wang

**Affiliations:** 1 Key Laboratory of Marine Drugs, The Ministry of Education of China, School of Medicine and Pharmacy, Ocean University of China, Qingdao 266003, China; dieying0719@163.com (M.C.); mayrush007@sina.com (X.-D.W.); ouc_zhaoqing@163.com (Q.Z.); 2 Marine Science & Technology Institute, College of Environmental Science & Engineering, Yangzhou University, 196#, Huayang West Street, Yangzhou 225127, China

**Keywords:** marine sponge, *Topsentia* sp., polyhydroxylated sterol, topsensterol, cytotoxicity

## Abstract

Three new polyhydroxylated sterol derivatives topsensterols A–C (**1**–**3**) have been isolated from a marine sponge *Topsentia* sp. collected from the South China Sea. Their structures were elucidated by detailed analysis of the spectroscopic data, especially the NOESY spectra. Topsensterols A–C (**l**–**3**) possess novel 2β,3α,4β,6α-tetrahydroxy-14α-methyl *Δ*^9(11)^ steroidal nuclei with unusual side chains. Compound **2** exhibited cytotoxicity against human gastric carcinoma cell line SGC-7901 with an IC_50_ value of 8.0 μM. Compound **3** displayed cytotoxicity against human erythroleukemia cell line K562 with an IC_50_ value of 6.0 μM.

## 1. Introduction

Most marine invertebrates are soft bodied and have a sedentary life style, resulting in the formation of distinctive chemical protection strategy [1,2]. The chemical means of defense necessitate the production of toxic compounds that can deter predators and paralyze their prey [2]. Undoubtedly, marine invertebrates represent a rich source of structurally novel and mechanistically unique secondary metabolites for the discovery of new drug leads [3]. Especially, sponges (Porifera) including the genus of *Topsentia* have been recognized as outstanding producers of sterols possessing novel side chains with unique alkylation and dealkylation patterns and displaying a variety of biological activities [4,5,6,7].

**Figure 1:**
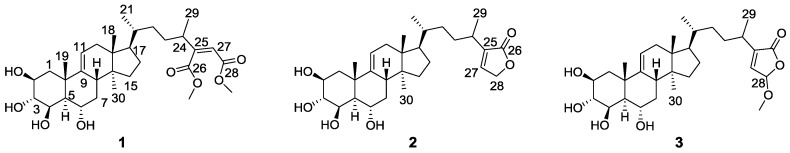
Chemical structures of compounds **1**–**3** (relative configuration).

In our continuing efforts to discover new bioactive substances from marine invertebrates, a chemical investigation of a sponge of *Topsentia* sp. collected from the South China Sea was carried out. As a result, three new polyhydroxylated steroids, topsensterols A–C (**1**–**3**) (Figure 1), were isolated from its *n*-butanol extract. Compounds **2** and **3** showed significant cytotoxicity against human gastric carcinoma cell line SGC-7901 and human erythroleukemia cell line K562, respectively. Herein, we report the isolation, structural elucidation, plausible biogenetic pathway and biological evaluation of these steroids.

## 2. Results and Discussion

The frozen sample of sponge *Topsentia* sp. (5.1 kg, wet weight) was exhaustively extracted with 95% EtOH (8 L × 5 times) at room temperature, and the EtOH solution was evaporated under reduced pressure to give the crude extract. This extract was further partitioned between H_2_O and EtOAc. After evaporation of the organic solvents, the remaining aqueous suspension was extracted with *n*-butanol. Then the organic layer was concentrated to offer the *n*-butanol extract (27.1 g). This extract was subjected to a silica gel column chromatograph (CC) and the fractions were purified by octadecylsilyl silica gel CC and semi-preparative HPLC to obtain compounds **1**–**3**.

**Figure 2:**
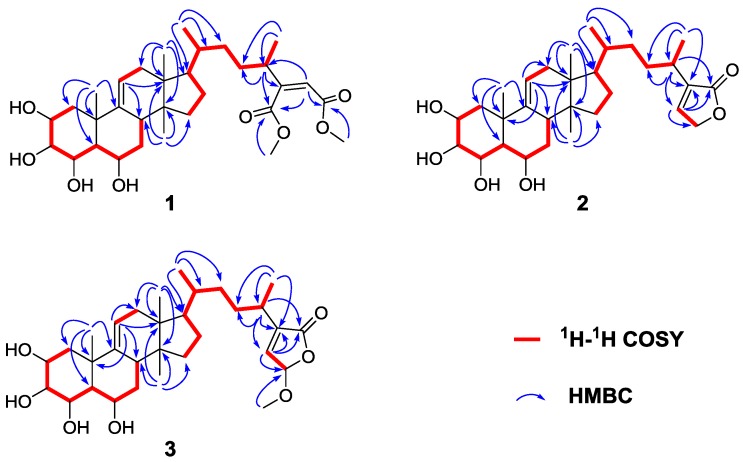
^1^H-^1^H COSY and key HMBC correlations of compounds **1**–**3**.

Topsensterol A (**1**) was obtained as a white amorphous powder. Its molecular formula was determined as C_32_H_50_O_8_ on the basis of HR-ESI-MS (Supplementary Materials, Figure S9), indicating 8 degrees of unsaturation. The ^1^H NMR spectrum (Table 1, Supplementary Materials, Figure S1) showed five methyl singlets including two methoxy signals (δ_H_ 3.70, 3.66, 1.34, 0.82, and 0.70) and two methyl doublets (δ_H_ 1.12 and 0.91) together with four oxygenated methine signals (δ_H_ 4.25, 4.11, 3.98, and 3.91). In addition, two olefinic signals (δ_H_ 5.86 (s), 5.32 (d, *J* = 5.2 Hz)) were observed. The ^13^C NMR spectrum (Table 2, Supplementary Materials, Figure S2) exhibited two ester carbonyls, two trisubstituted double bonds, seven methyls, seven methylenes, nine methines including four oxygenated methines, and three quaternary sp^3^ carbons. Therefore, a steroid nature was suggested for this molecule. The HMBC correlations (Figure 2, Supplementary Materials, Figure S5) observed from Me-30 to C-8, C-14, and C-15 demonstrated that a methyl group anchored at C-14 of the steroid nucleus. Interpretation of the COSY correlations (Figure 2, Supplementary Materials, Figure S4) from H-1 to H-8 indicated a contiguous sequence including four oxygenated methines, assigning four hydroxy groups were at C-2, C-3, C-4, and C-6, respectively. The COSY crosspeaks of H-11/H-12 and HMBC correlations from H-11 to C-8, C-10, and C-13 inferred the *Δ*^9(11)^-unsaturated structure in steroid nucleus. The above spectroscopic data indicated that **1** belongs to 14α-methyl *Δ*^9(11)^-unsaturated steroids previously isolated from marine sponge, *Topsentia* sp. [8]. In addition, HMBC correlations from 26-OMe to C-26 and from 28-OMe to C-28 showed 26-OMe and 28-OMe were connected to C-26 and C-28, respectively. HMBC correlations from H-27 to C-24, C-25, C-26, and C-28, and from H-24 to C-23, C-25, and C-26 revealed the double bond was between C-25 and C-27. Consequently, a 2-substituted-dimethyl maleate unit was suggested in the side chain. Therefore the planar structure of **1** was established.

**Figure 3:**
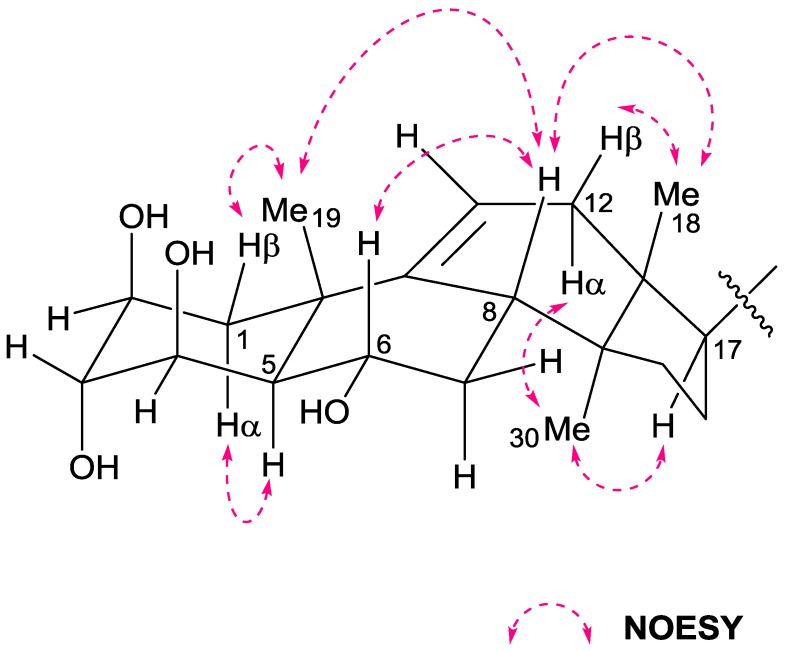
Key NOESY correlations for the sterol nucleus of compound **1**.

The stereochemistry of the steroid nucleus was established on the basis of coupling constants, COSY and NOESY data. Small coupling constants between H-2 and both H_2_-1 suggested that H-2 adopted an equatorial orientation. A large coupling constant between H-5 and H-6 (*J* = 10.8 Hz) and a small coupling constant between H-5 and H-4 (*J* = 2.4 Hz), implied that both H-5 and H-6 were axial, and H-4 was equatorial. In the COSY spectrum, a conspicuous *W*-type long-range crosspeaks between H-2 and H-4 further confirmed both H-2 and H-4 were equatorial. While *W*-type long-range crosspeaks between H-lβ and H-3 indicated that H-3 occupied an equatorial orientation. Therefore, the hydroxyl groups 2-OH, 3-OH, 4-OH, and 6-OH occupied the axial, axial, axial, and equatorial orientation, respectively. In the NOESY spectrum (Figure 3, Supplementary Materials, Figures S6 and S7), the absence of a crosspeak between Me-19 and H-5 suggested a trans-fused A/B ring junction. The NOESY crosspeaks of Me-19/H-8, Me-18/H-8, Me-18/H-12β, and H-6/H-8 indicated these protons were β-orientation, while crosspeaks of Me-30/H-17, and Me-30/H-12α revealed these protons were α-orientation. Furthermore, NOESY crosspeaks observed between Me-29 and H-27 suggested the double bond occupied *Z*-configuration, confirming the presence of a 2-substituted-dimethyl maleate unit in **1**.

Careful inspection of the NMR spectra of **1**–**3** (Table 1 and Table 2) showed that the common and highly conserved signals of the steroidal nuclei for **1**–**3** are strikingly similar, including the signals of three angular methyl groups, a C-9(11)-double bond, four oxygenated methines, and four hydroxyl groups. Combined with their NOESY data and comparison with the configurations of the known similar metabolites such as topsentiasterol sulfates A–F, chlorotopsentiasterol sulfate D, and iodotopsentiasterol sulfate D also isolated from the marine sponge *Topsentia* sp. [7,8], compounds **1**–**3** were suggested to possess the same 2β,3α,4β,6α-tetrahydroxy-14α-methyl *Δ*^9(11)^ sterol framework. The differences between these compounds were restricted to the respective aliphatic side chains.

Topsensterol B (**2**) was isolated as a white amorphous powder with a molecular formula of C_30_H_46_O_6_ determined on the basis of HR-ESI-MS (Supplementary Materials, Figure S16). The COSY spectrum (Supplementary Materials, Figure S13) revealed the connectivities of H_3_-21/H-20/H_2_-22/H_2_-23/H-24/H_3_-29 and the terminal γ-lactone in the side chain was deduced by interpretation of HMBC data (Supplementary Materials, Figure S14) together with NMR signals (Table 1 and Table 2, Supplementary Materials, Figures S10 and S11) for an oxymethylene (δ_H_ 4.87, δ_C_ 71.7), a trisubstituted double bond (δ_H_ 7.32, δ_C_ 139.6, 146.1) and an ester carbonyl (δ_C_ 176.3). These NMR signals also corresponded well with those reported for 2-alkyl butenolide [8].

Topsensterol C (**3**), with molecular formula of C_31_H_48_O_7_, was also isolated as a white amorphous powder. The ^1^H and ^13^C NMR signals (Table 1 and Table 2, Supplementary Materials, Figures S17 and S18) of the side chain were very similar to those of **2**. The only significant difference was that a methoxy (δ_H_ 3.58, δ_C_ 57.0) and an oxygenated methine (δ_H_ 5.87, δ_C_ 104.0) in **3** replaced the oxymethylene (δ_H_ 4.87, δ_C_ 71.7) in **2**. Interpretation of HMBC data (Supplementary Materials, Figure S21) of **3** indicated that a 4-methoxy-2-alkyl butenolide moiety connected at the terminal of the side chain for **3**.

**Scheme 1:**
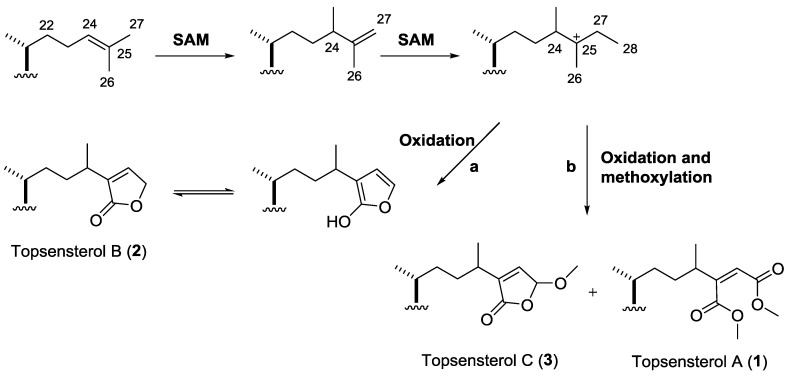
Plausible biosynthesis mechanism to form the side chains of **1**–**3**.

Polyhydroxylated sterols with various novel side chains are common secondary metabolites from marine sponges. In present study, topsensterol A (**1**) possesses a unique side chain terminated with a 2-substituted-dimethyl maleate unit. To the best of our knowledge, it is the first report of polyhydroxylated sterol with a 2-substituted-dimethyl maleate side chain. The plausible biosynthesis mechanism to form the side chains of **1**–**3** was proposed in Scheme 1. According to the literature [6,7], the side chains present in **1**–**3** are likely formed via methylations by *S*-adenosylmethionine (SAM) to the side chain present in parkeol, along with oxidation and methoxylation. Scheme 1 illustrates that: (i) the addition of a methyl from SAM to C-24 and a loss of a proton to generate the 25,27-double bond; (ii) SAM adds a methyl to C-27 to form the C-25 cation; (iii) the C-25 cation followed by oxidation and tautomerization to form topsensterol B (**2**) (pathway **a**); and (iv) the C-25 cation followed by oxidation and methoxylation to form topsensterol C (**3**) and topsensterol A (**1**) (pathway **b**).

Marine sponges of the genus *Topsentia* were reported to produce various structurally unique steroids including polyhydroxylated sterols [9] and polysulfated steroids [10,11,12]. In the present study, all of the isolated polar steroids were polyhydroxylated sterols, as the desulfated derivatives of similar sterol sulfates [8]. It seems that our isolated compounds maybe a group of desulfated artifacts. However, the desulfation of polysulfated sterols should be not so easy to occur during the ordinary extraction and separation processes. To date many polysulfated sterols have been reported to be obtained from marine sponges via ordinary separation processes, indicating that polysulfated sterols are stable enough. More importantly, on the basis of biogenetic considerations, the 2β,3α,4β,6α-tetrahydroxy-14α-methyl *Δ*^9(11)^ steroidal nuclei pattern could be biosynthesized directly from parkeol in marine sponges [6,13,14]. Given the above, compounds **1**–**3** were more likely to be produced by ecological conditions, but the probability of desulfated artifacts could not be ruled out.

Compounds **1**–**3** were assessed for their cytotoxic activity against human gastric carcinoma SGC-7901 and human erythroleukemia K562 cell lines by MTT method [15]. Compound **2** exhibited cytotoxicity against SGC-7901 and K562 cell lines with IC_50_ values of 8.0 and 20 μM, respectively. Compound **3** exhibited cytotoxicity against SGC-7901 and K562 cell lines with IC_50_ values of 28 and 6.0 μM, respectively. However, compound **1** showed no cytotoxicity against the two cell lines. The above results revealed that the terminal butenolide moiety in the side chain may play a key role in the cytotoxicity.

Compounds **1**–**3** were also tested for their antimicrobial activity against human pathogenic bacteria including *Staphylococcus aureus* (ATCC 51650), Methicillin-resistant *Staphylococcus aureus* (ATCC 9551), and *Candida albicans* (ATCC 10231) using the method developed by Fromtling et al. [16]. However, no compound was found to be active against these bacteria.

## 3. Experimental Section

### 3.1. General Experimental Procedures

Optical rotation values were measured on a Jasco-P-1020 digital polarimeter (Jasco Corp., Tokyo, Japan). NMR spectra were recorded on a Bruker AV-400 NMR spectrometer (Bruker Corp., Fallanden, Switzerland), and chemical shifts were recorded as δ values (400 MHz for ^1^H and 100 MHz for ^13^C). ESIMS spectra were obtained from a Micromass Q-TOF spectrometer (Waters Corp., Milford, MA, USA). Semipreparative HPLC was performed on a Waters 1525 system using a semipreparative C_18_ column (5 μm, 10 × 250 mm, Kromasil, Sweden) coupled with a Waters 2996 photodiode array detector (Waters Corp., Milford, MA, USA). Silica gel (200–300 mesh, Qingdao Haiyang Chemical Factory, Qingdao, China), and octadecylsilyl (ODS) silica gel (45–60 mm; Merck KGaA, Darmstadt, Germany) were used for column chromatography. Thin-layer chromatography (TLC) was performed on precoated silica gel 60 GF254 plates (Yantai Zifu Chemical Group Co., Yantai, China).

### 3.2. Animal Material

The marine sponge *Topsentia* sp. was collected from Xuwen, Guangdong Province, China, in April 2006, and was identified by Nicole J. de Voogd, National Museum of Natural History, The Netherlands. A voucher specimen (GD-XW-20060007) was deposited at the Key Laboratory of Marine Drugs, Ministry of Education, Ocean University of China, Qingdao, China.

### 3.3. Extraction and Isolation

The *n*-butanol extract (27.1 g) was subjected to a silica gel column chromatograph (CC) and eluted with a gradient of CHCl_3_/MeOH (50:1–0:1, *v*:*v*) to collect 3 fractions (Fr.A–Fr.C). Fr.A displayed cytotoxicity and the ^1^H NMR features of diverse sterol analogues. This fraction was fractionated upon a octadecylsilyl silica gel CC and eluted with a gradient of H_2_O/MeOH (50:1–0:1, *v*:*v*) to obtain three subfractions (Fr.A1–Fr.A3). Fr.A1 was subjected to semi-preparative HPLC eluted with MeOH/H_2_O (80/20, *v*/*v*) to obtain compounds **2** (4.2 mg) and **3** (6.4 mg). Fr.A2 was subjected to semi-preparative HPLC eluted with MeOH/H_2_O (85/15, *v*/*v*) to yield compound **1** (2.8 mg).

Topsensterol A (**1**): white amorphous powder; [α]${}_{D}^{20}$ +11 (*c* 0.1, MeOH); for ^1^H and ^13^C NMR data, see Table 1 and Table 2; HRESIMS *m*/*z* 563.3600 [M + H]^+^ (calcd. for C_32_H_51_O_8_^+^, 563.3584).

Topsensterol B (**2**): white amorphous powder; [α]${}_{D}^{20}$ +14 (*c* 0.1, MeOH); for ^1^H and ^13^C NMR data, see Table 1 and Table 2; HRESIMS *m*/*z* 520.3621 [M + NH_4_]^+^ (calcd. for C_30_H_50_NO_6_^+^, 520.3633).

Topsensterol C (**3**): white amorphous powder; [α]${}_{D}^{20}$ +17 (*c* 0.1, MeOH); for ^1^H and ^13^C NMR data, see Table 1 and Table 2; HRESIMS *m*/*z* 550.3732 [M + NH_4_]^+^ (calcd. for C_31_H_52_NO_7_^+^, 550.3738).

## 4. Conclusions

In summary, three new polyhydroxylated steroid derivatives topsensterols A–C (**l**–**3**) possessing unusual side chains were successfully isolated from the marine sponge *Topsentia* sp. The plausible biosynthesis mechanism to form the uncommon side chains of **1**–**3** was proposed. Compounds **2** and **3** exhibited significant cytotoxicities, demonstrating that the terminal butenolide moiety in the side chain may act an important part in the cytotoxicity.

## Figures and Tables

**Table 1:** ^1^H NMR spectroscopic data (400 MHz, CD_3_OD) for **1**–**3**.

H	1	2	3
1α	1.96 (dd, 13.0, 4.8)	2.00 (m)	1.98 (m)
1β	2.40 (m)	2.46 (br d, 13.0)	2.46 (br d, 14.0)
2	4.25 (br s)	4.28 (br s)	4.28 (br s)
3	3.91 (t, 2.4)	3.98 (t, 2.8)	3.97 (t, 3.2)
4	3.98 (br d, 2.4)	4.04 (br d, 2.8)	4.03 (br d, 3.2)
5	1.41 (dd, 10.8, 2.0)	1.46 (dd, 10.8, 2.0)	1.45 (dd, 10.8, 2.0)
6	4.11 (td, 10.8, 4.4)	4.16 (td, 10.8, 4.4)	4.15 (td, 10.8, 4.4)
7α	1.51 (m)	1.53 (m)	1.53 (m)
7β	1.91 (m)	1.91 (m)	1.91 (m)
8	1.95 (m)	1.95 (m)	1.95 (m)
11	5.32 (d, 5.2)	5.36 (d, 5.2)	5.36 (br d, 5.2)
12α	2.12 (d, 17.5)	2.15 (br d, 17.5)	2.15 (br d, 17.5)
12β	1.93 (m)	1.93 (m)	1.93 (m)
15a	1.38 (m)	1.38 (m)	1.38 (m)
15b	1.44 (m)	1.44 (m)	1.44 (m)
16a	1.37 (m)	1.37 (m)	1.37 (m)
16b	1.92 (m)	1.92 (m)	1.92 (m)
17	1.66 (q, 9.4)	1.69 (q, 9.4)	1.68 (q, 9.4)
18	0.70 (s)	0.73 (s)	0.73 (s)
19	1.34 (s)	1.38 (s)	1.38 (s)
20	1.42 (m)	1.42 (m)	1.42 (m)
21	0.91 (d, 6.4)	0.95 (d, 6.4)	0.95 (d, 6.4)
22a	1.06 (m)	1.11 (m)	1.11 (m)
22b	1.43 (m)	1.48 (m)	1.48 (m)
23a	1.42 (m)	1.47 (m)	1.47 (m)
23b	1.54 (m)	1.55 (m)	1.55 (m)
24	2.42 (m)	2.52 (q, 6.4)	2.53 (q, 6.4)
27	5.86 (s)	7.32 (br s)	6.93 (br s)
28		4.87 (br s)	5.87 (br s)
29	1.12 (d, 6.8)	1.21 (d, 6.8)	1.20 (d, 6.8)
30	0.82 (s)	0.85 (s)	0.85 (s)
26-OCH_3_	3.70 (s)		
28-OCH_3_	3.66 (s)		3.58 (s)

**Table 2:** ^13^C NMR spectroscopic data (100 MHz, CD_3_OD) for **1**–**3**.

C	1	2	3
1	39.9, CH_2_	39.7, CH_2_	39.8, CH_2_
2	71.5, CH	71.2, CH	71.3, CH
3	72.5, CH	72.0, CH	72.2, CH
4	72.8, CH	72.4, CH	72.6, CH
5	48.1, CH	48.8, CH	49.0, CH
6	66.7, CH	66.4, CH	66.5, CH
7	37.9, CH_2_	37.5, CH_2_	37.6, CH_2_
8	41.4, CH	41.0, CH	41.1, CH
9	147.9, C	147.4, C	147.5, C
10	39.8, C	39.5, C	39.5, C
11	117.0, CH	116.9, CH	116.9, CH
12	38.4, CH_2_	38.1, CH_2_	38.1, CH_2_
13	45.7, C	45.4, C	45.4, C
14	48.1, C	47.8, C	47.9, C
15	34.9, CH_2_	34.7, CH_2_	34.7, CH_2_
16	29.0, CH_2_	28.8, CH_2_	28.8, CH_2_
17	52.3, CH	51.9, CH	51.9, CH
18	15.1, CH_3_	15.1, CH_3_	15.1, CH_3_
19	27.1, CH_3_	27.0, CH_3_	27.0, CH_3_
20	37.5, CH	37.0, CH	37.1, CH
21	18.9, CH_3_	18.9, CH_3_	18.9, CH_3_
22	34.7, CH_2_	34.4, CH_2_	34.5, CH_2_
23	32.7, CH_2_	32.5, CH_2_	32.4, CH_2_
24	40.5, CH	31.8, CH	31.9, CH
25	157.6, C	139.6, C	144.2, C
26	170.7, C	176.3, C	172.8, C
27	119.4, CH	146.1, CH	143.1, CH
28	167.1, C	71.7, CH_2_	104.0, CH
29	19.3, CH_3_	18.8, CH_3_	18.8, CH_3_
30	19.0, CH_3_	18.9, CH_3_	18.9, CH_3_
26-OCH_3_	52.3, CH_3_		
28-OCH_3_	52.7, CH_3_		57.0, CH_3_

## Supplementary Materials

The following are available online at www.mdpi.com/1660-3397/14/8/146/s1, HRESIMS, 1D and 2D NMR data of compounds **l**–**3**.
