# Supplementary material for: Topsensterols A–C, Cytotoxic Polyhydroxylated Sterol Derivatives from a Marine Sponge Topsentia sp
Source: Mar Drugs. 2016 Aug 1;14(8):146. doi: 10.3390/md14080146 (PMC4999907; doi:10.3390/md14080146)
Supplement: Supplementary file 1 [file marinedrugs-14-00146-s001.pdf]

Supplementary Materials: Topsensterols A–C, Cytotoxic Polyhydroxylated Sterol Derivatives from a Marine Sponge *Topsentia* sp.

Min Chen, Xu-Dong Wu, Qing Zhao and Chang-Yun Wang

List of Supplementary Materials

Figure S1. ^{1}H NMR (400 MHz, CD_3OD) spectrum of compound **1**
Figure S2. ^{13}C NMR and DEPT (100 MHz, CD_3OD) spectra of compound **1**
Figure S3. HSQC (CD_3OD) spectrum of compound **1**
Figure S4. ^{1}H–^{1}H COSY (CD_3OD) spectrum of compound **1**
Figure S5. HMBC (CD_3OD) spectrum of compound **1**
Figure S6. NOESY (CD_3OD) spectrum of compound **1**
Figure S7. Partial NOESY (CD_3OD) spectrum of compound **1**
Figure S8. ESIMS spectrum of compound **1**
Figure S9. HRESIMS spectrum of compound **1**
Figure S10. ^{1}H NMR (400 MHz, CD_3OD) spectrum of compound **2**
Figure S11. ^{13}C NMR and DEPT (100 MHz, CD_3OD) spectra of compound **2**
Figure S12. HSQC (CD_3OD) spectrum of compound **2**
Figure S13. ^{1}H–^{1}H COSY (CD_3OD) spectrum of compound **2**
Figure S14. HMBC (CD_3OD) spectrum of compound **2**
Figure S15. NOESY (CD_3OD) spectrum of compound **2**
Figure S16. HRESIMS spectrum of compound **2**
Figure S17. ^{1}H NMR (400 MHz, CD_3OD) spectrum of compound **3**
Figure S18. ^{13}C NMR and DEPT (100 MHz, CD_3OD) spectra of compound **3**
Figure S19. HSQC (CD_3OD) spectrum of compound **3**
Figure S20. ^{1}H–^{1}H COSY (CD_3OD) spectrum of compound **3**
Figure S21. HMBC (CD_3OD) spectrum of compound **3**
Figure S22. HRESIMS spectrum of compound **3**

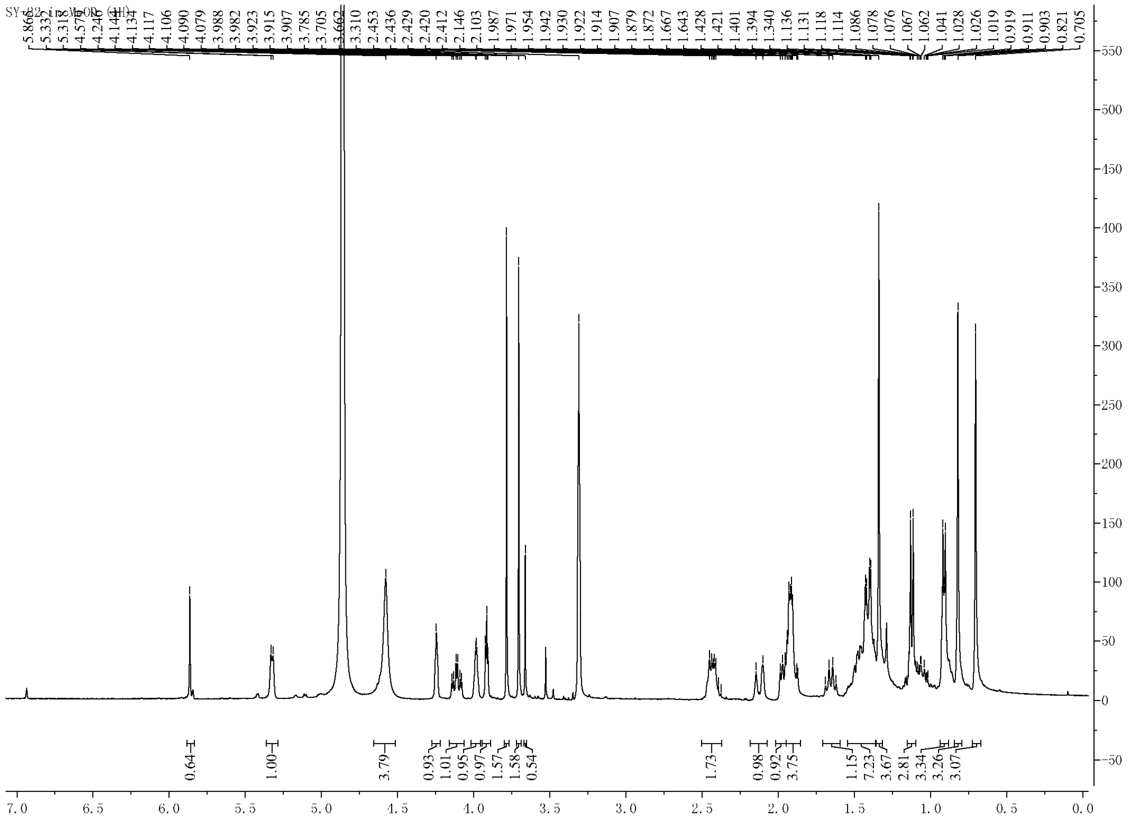

Figure S1. ^{1}H NMR (600 MHz, CD_3OD) spectrum of compound **1**.

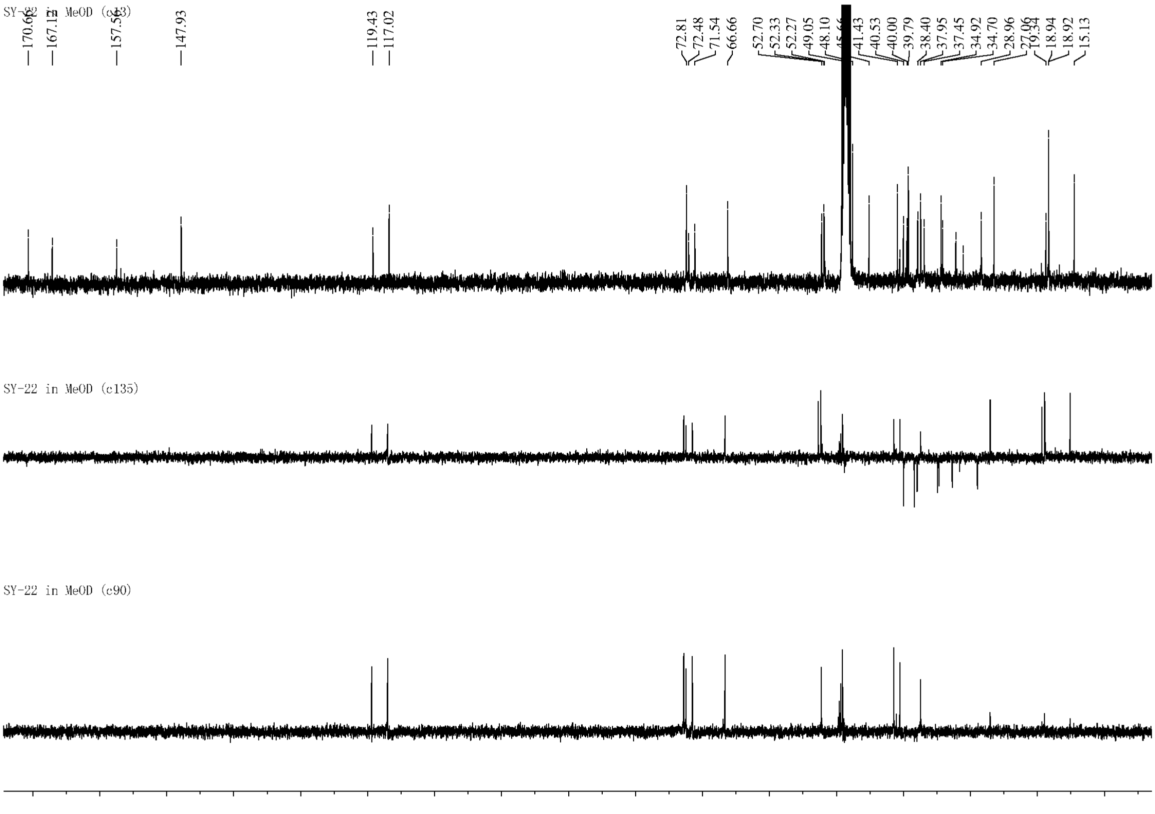

Figure S2. ^{13}C NMR and DEPT (100 MHz, CD_3OD) spectra of compound **1**.

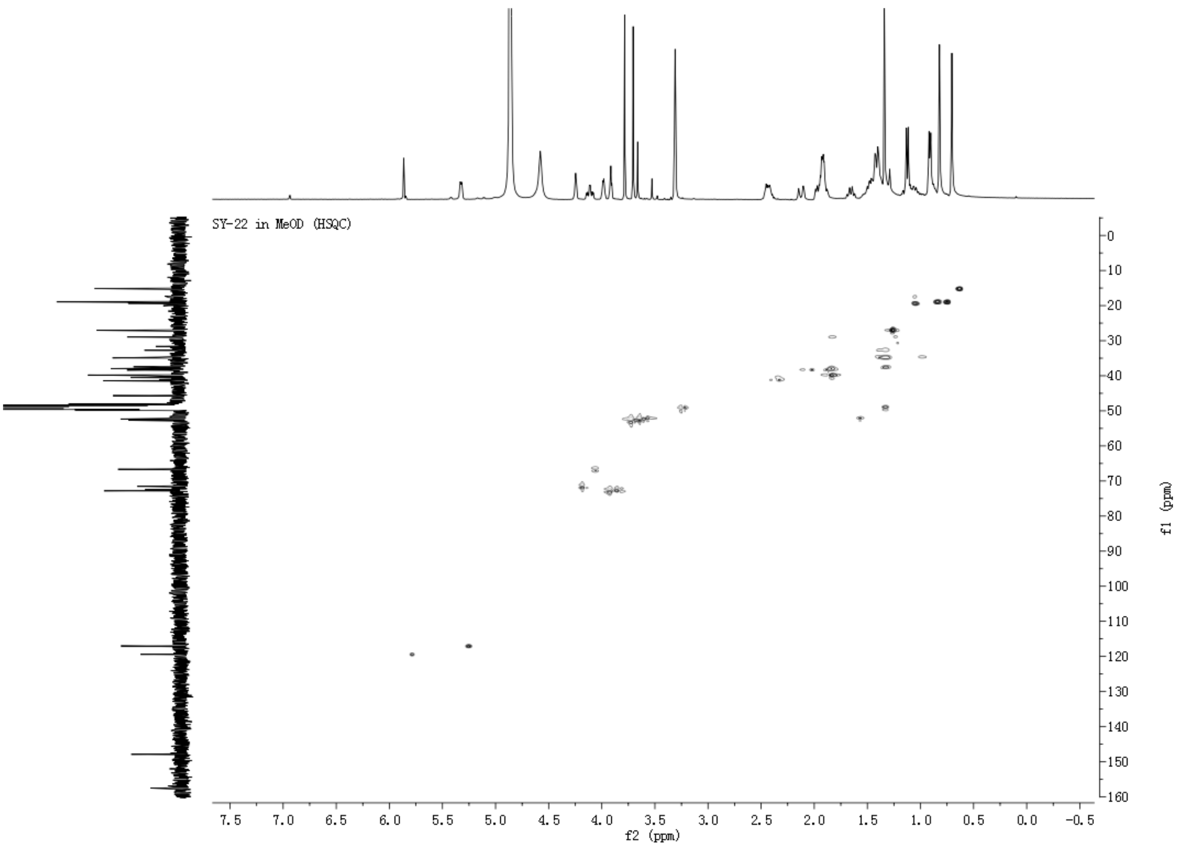

Figure S3. HSQC (CD_3OD) spectrum of compound **1**.

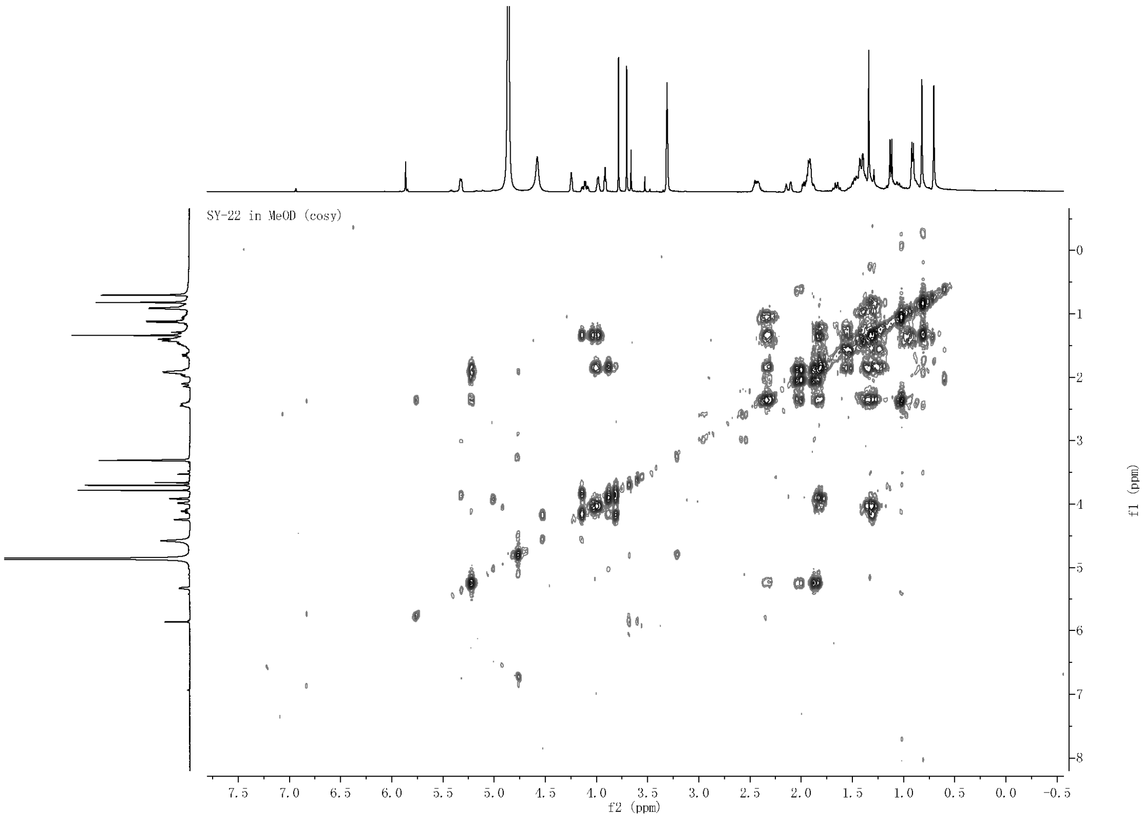

Figure S4. ^1H–^1H COSY (CD_3OD) spectrum of compound **1**.

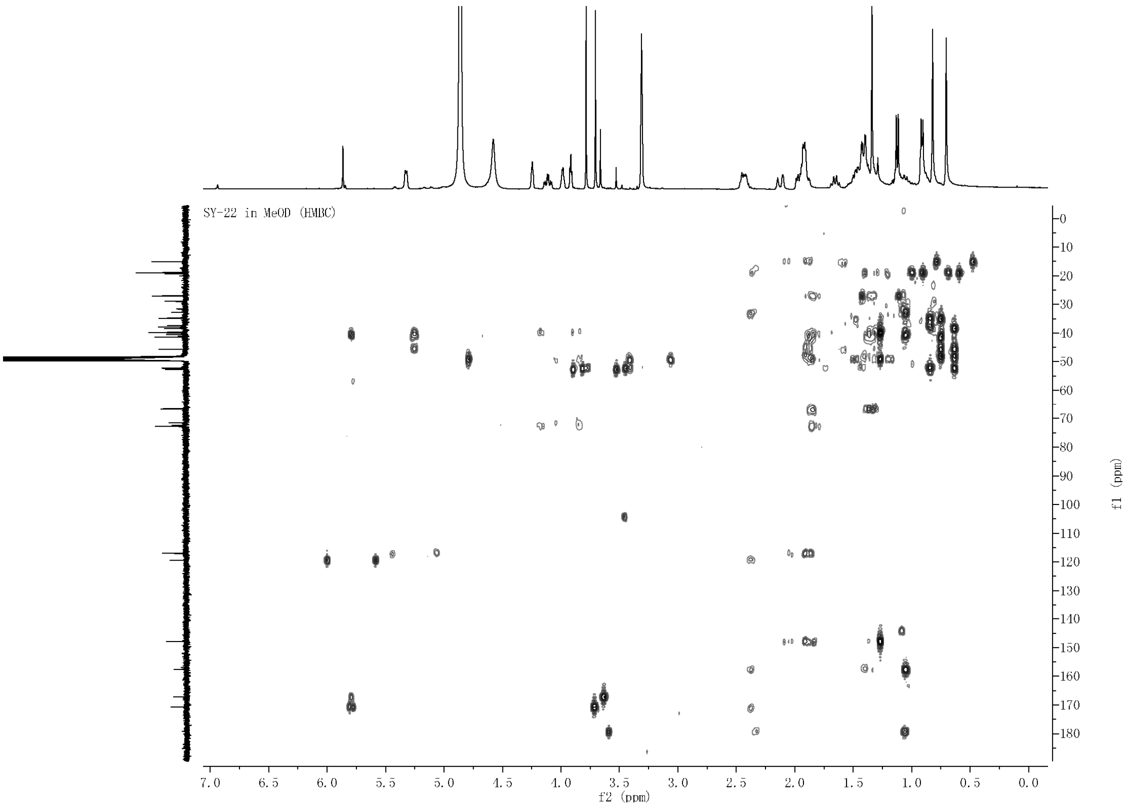

Figure S5. HMBC (CD_3OD) spectrum of compound **1**.

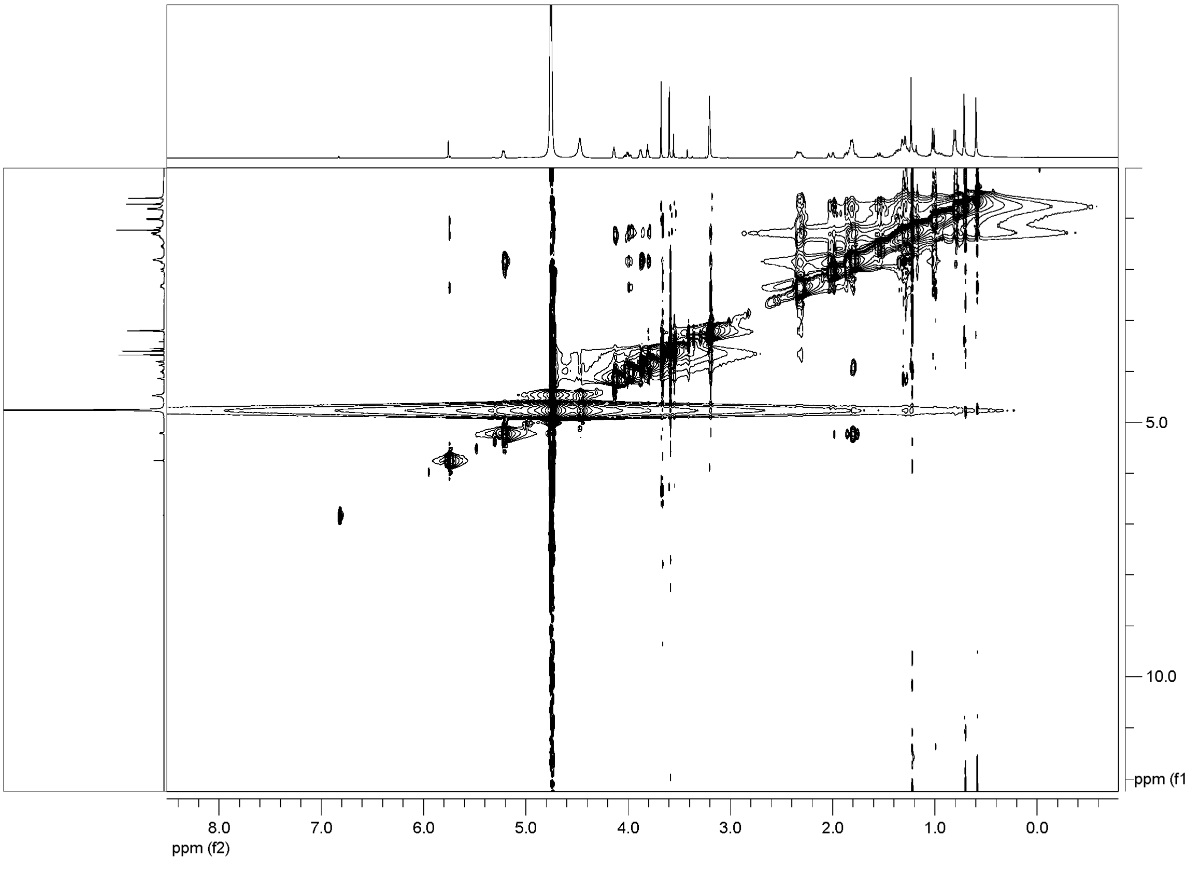

Figure S6. NOESY (CD_3OD) spectrum of compound **1**.

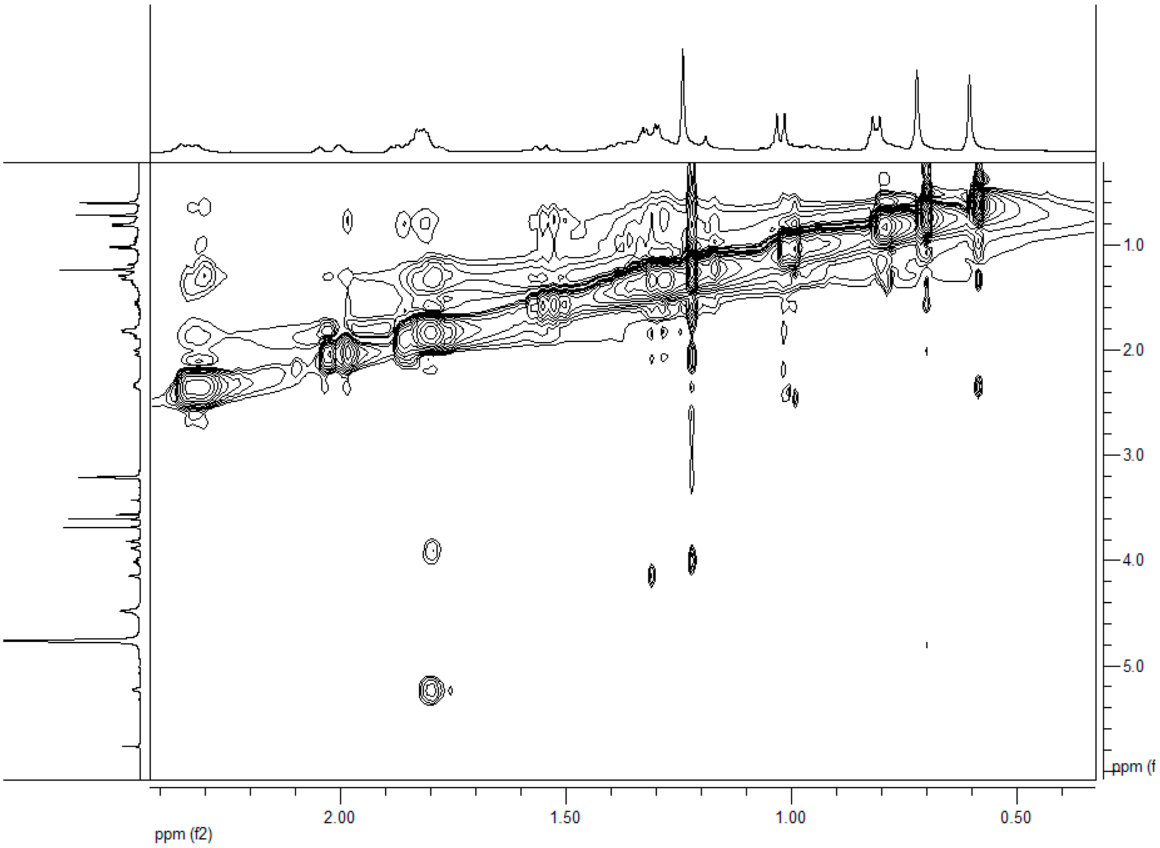

Figure S7. Partial NOESY (CD_3OD) spectrum of compound **1**.

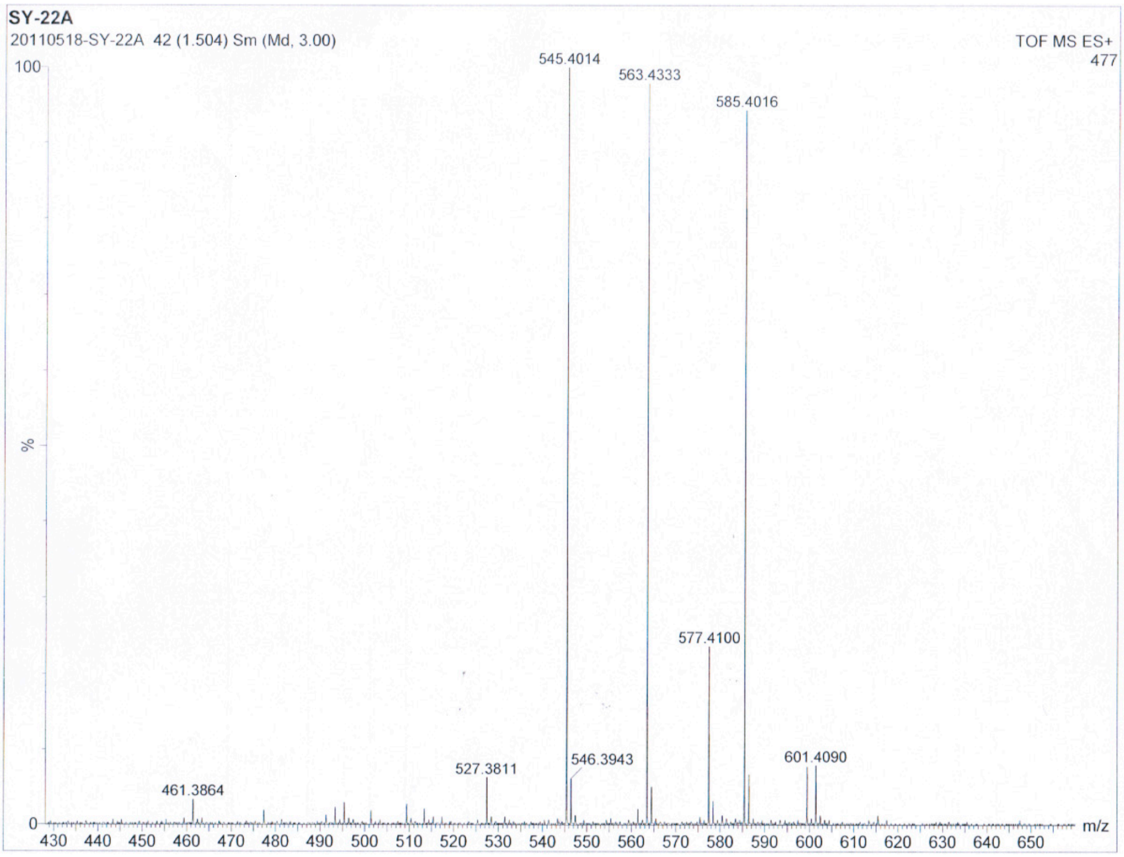

Figure S8. ESIMS spectrum of compound **1**.

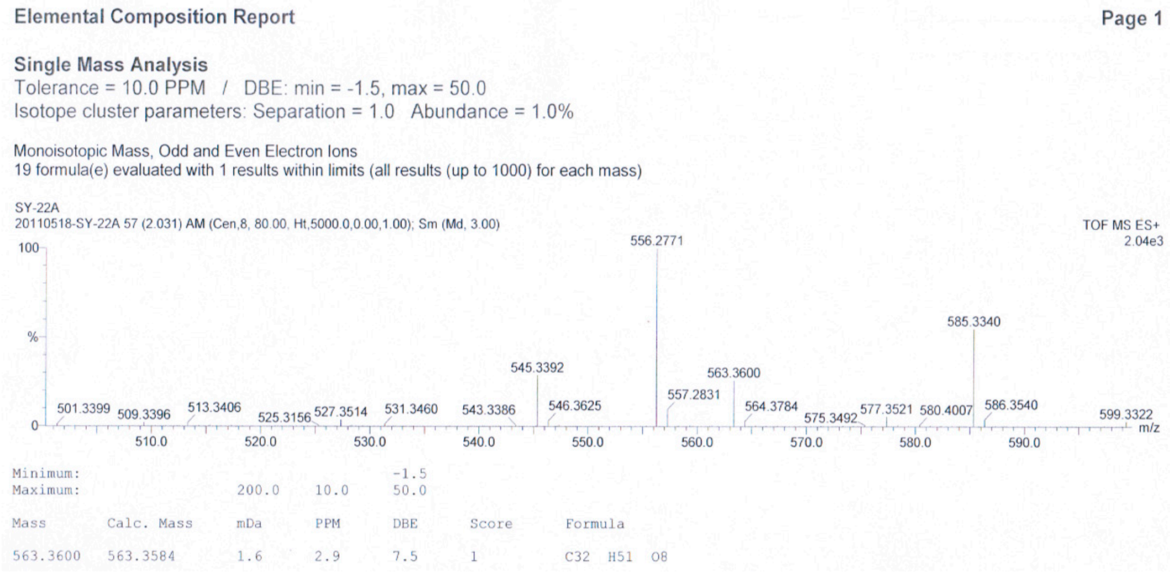

Figure S9. HRESIMS spectrum of compound **1**.

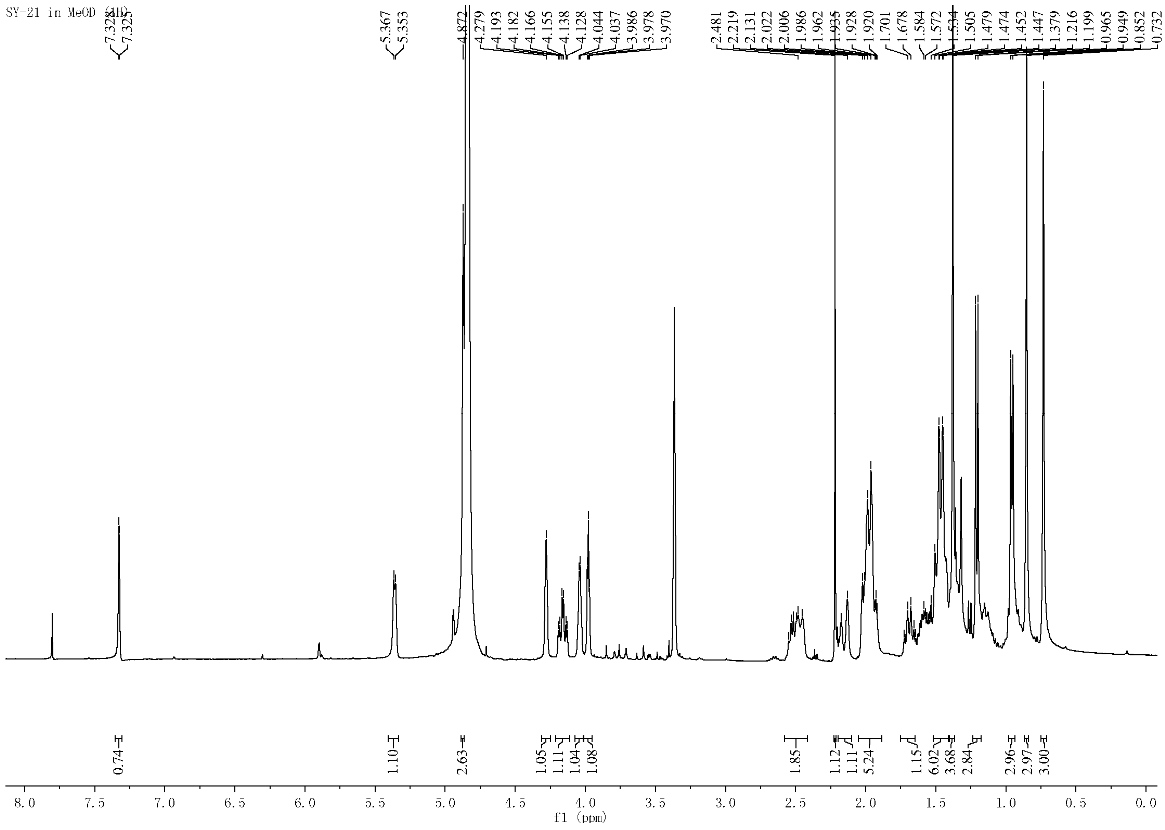

Figure S10. ^1H NMR (400 MHz, CD_3OD) spectrum of compound **2**.

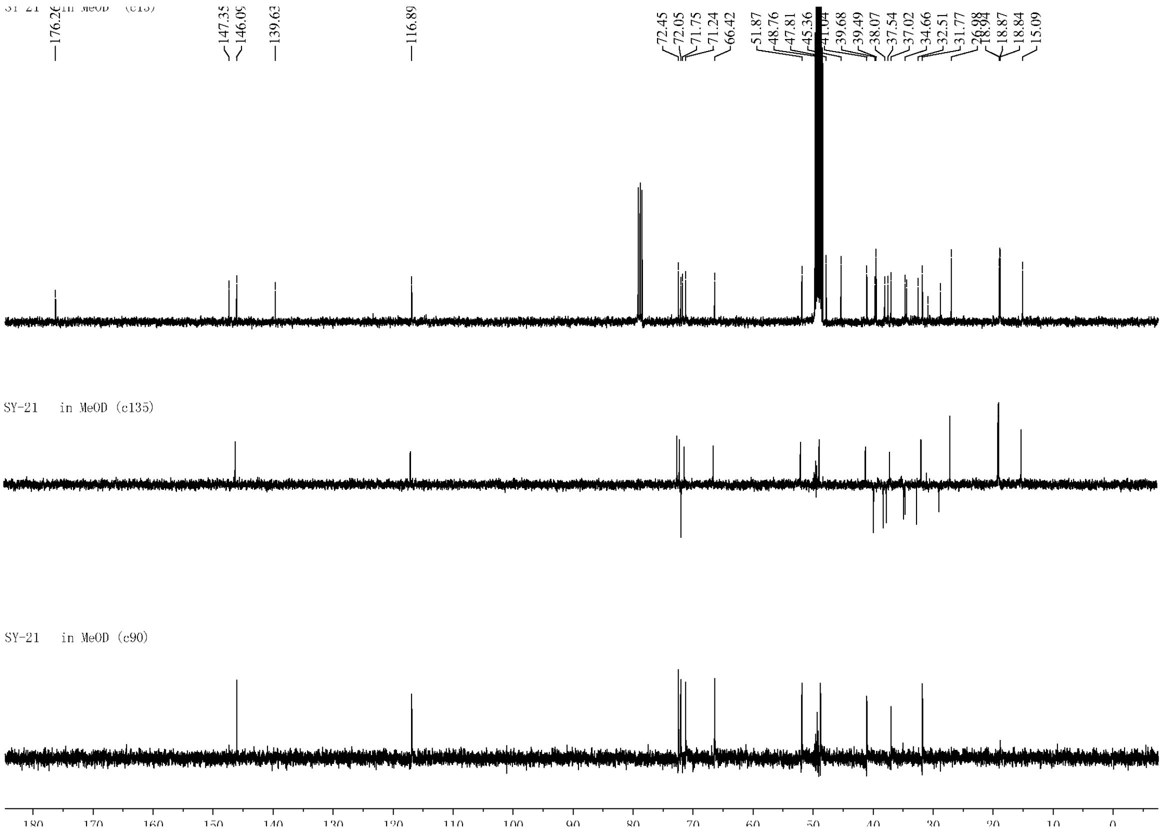

Figure S11. ^{13}C NMR and DEPT (100 MHz, CD_3OD) spectra of compound **2**.

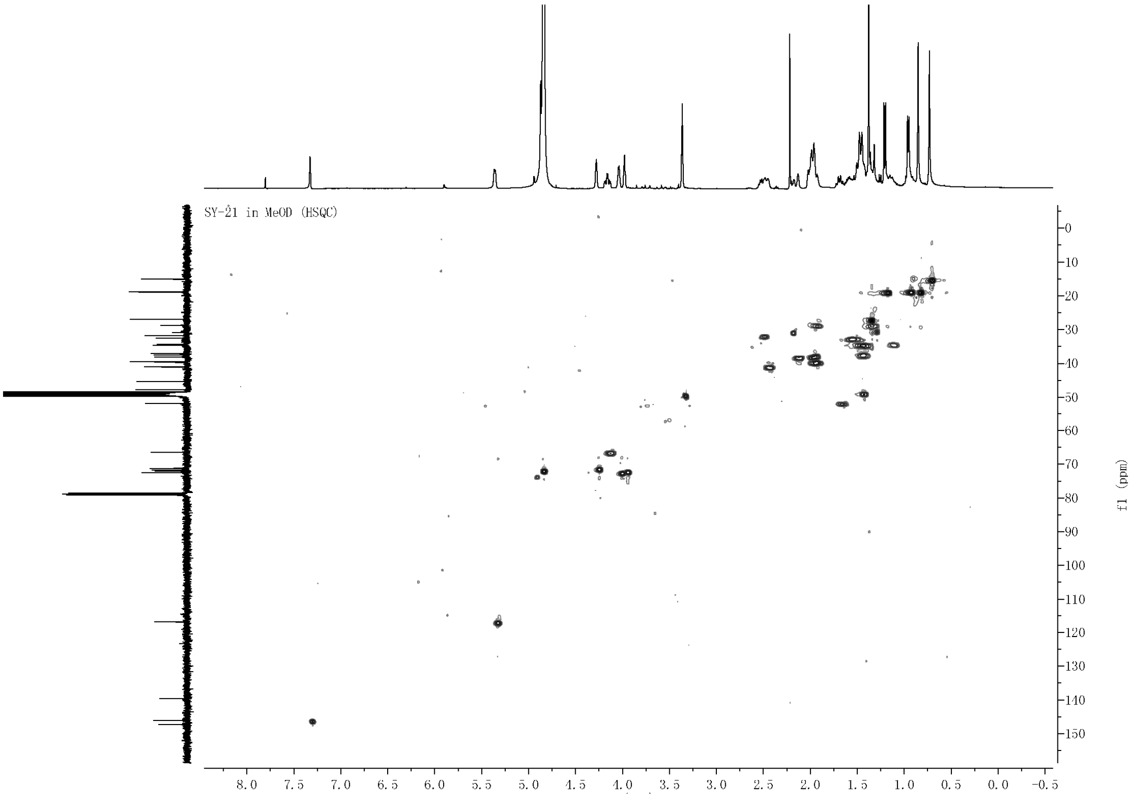

Figure S12. HSQC (CD_3OD) spectrum of compound **2**.

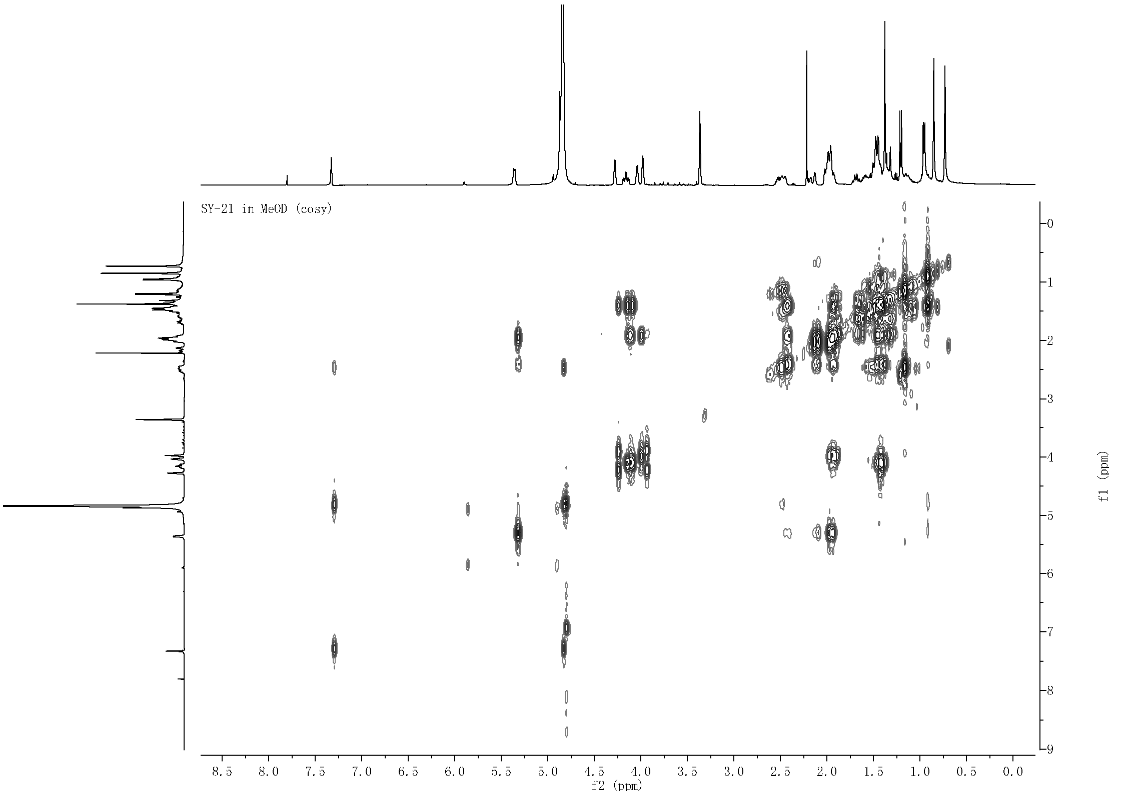

Figure S13. ^1H–^1H COSY (CD_3OD) spectrum of compound **2**.

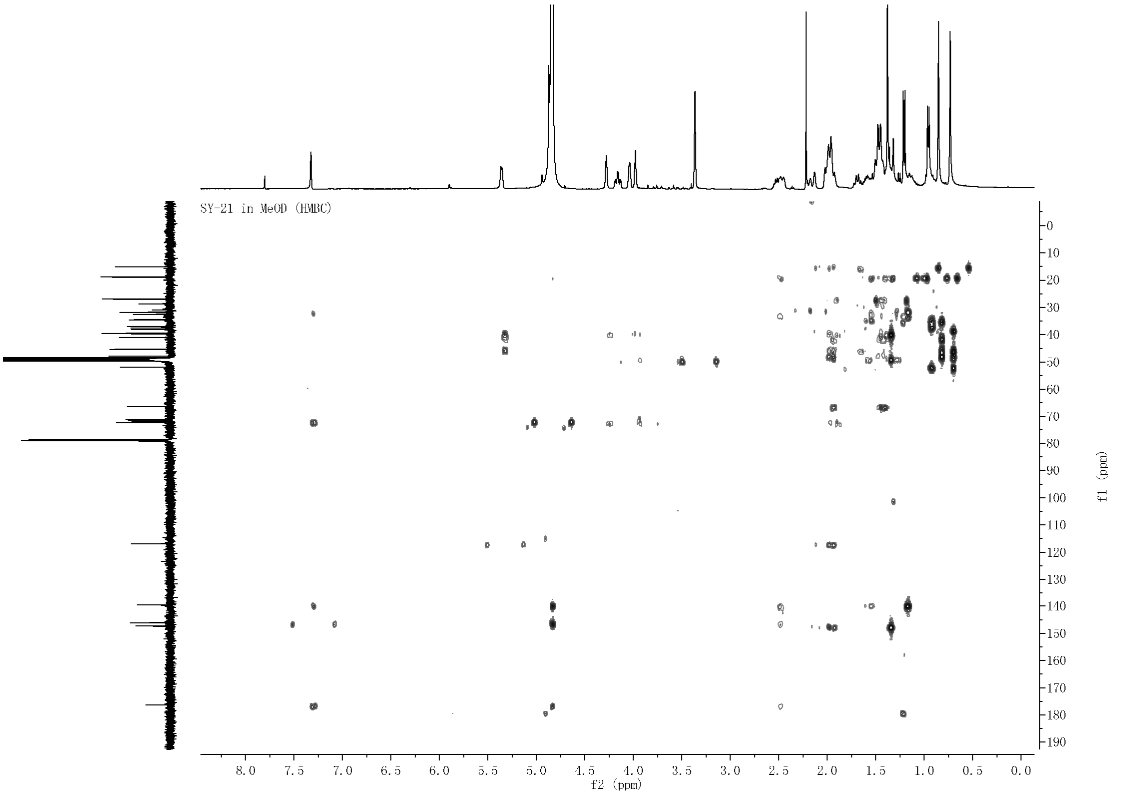

Figure S14. HMBC (CD_3OD) spectrum of compound **2**.

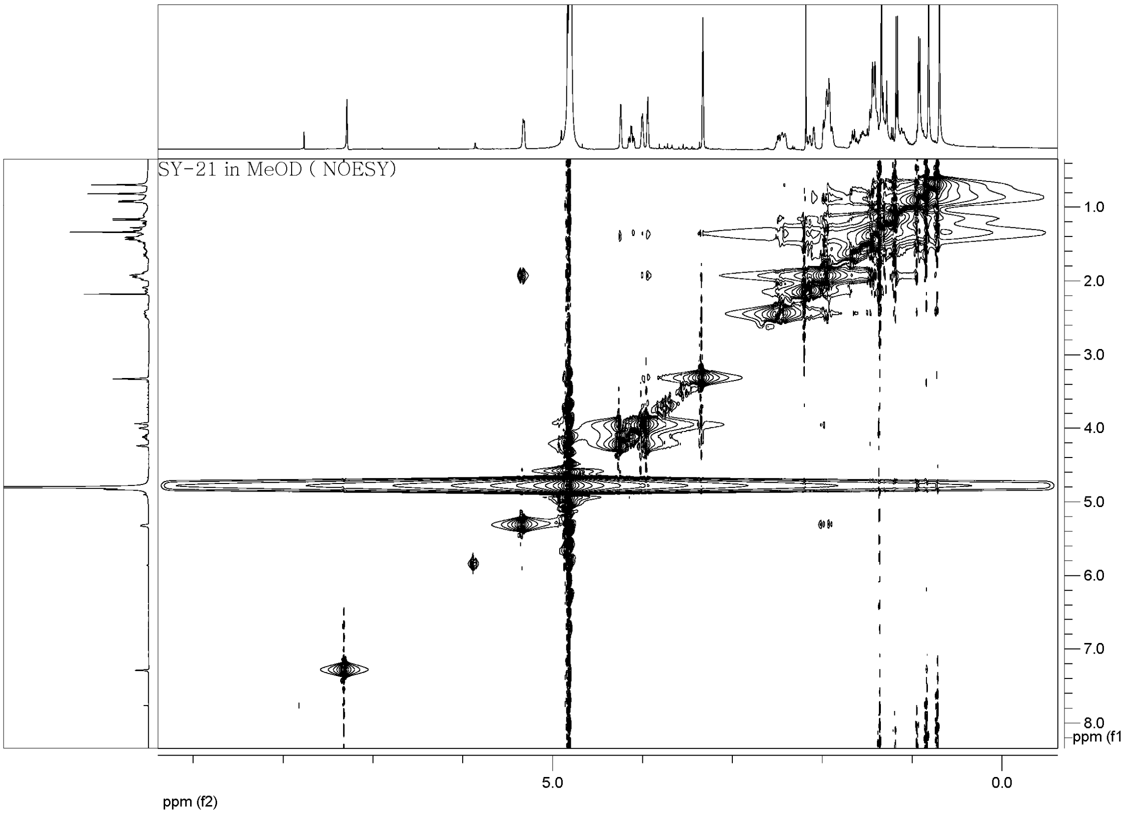

Figure S15. NOESY (CD_3OD) spectrum of compound **2**.

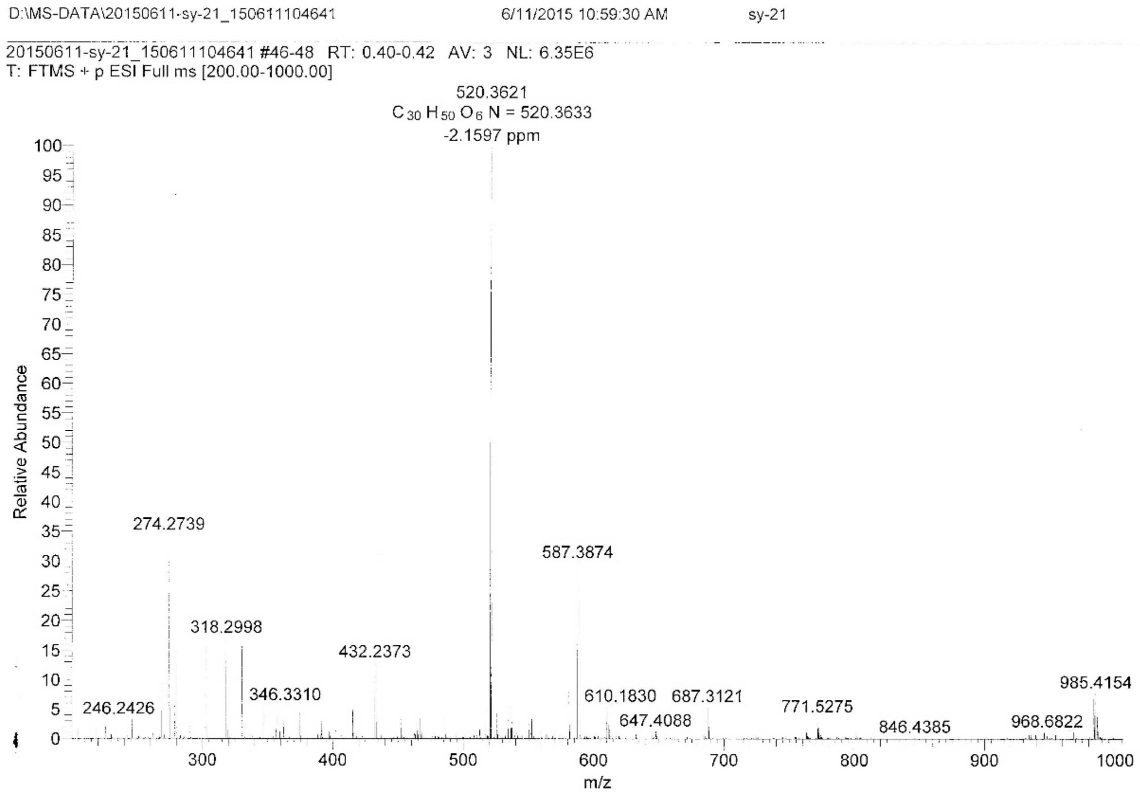

Figure S16. HRESIMS spectrum of compound **2**.

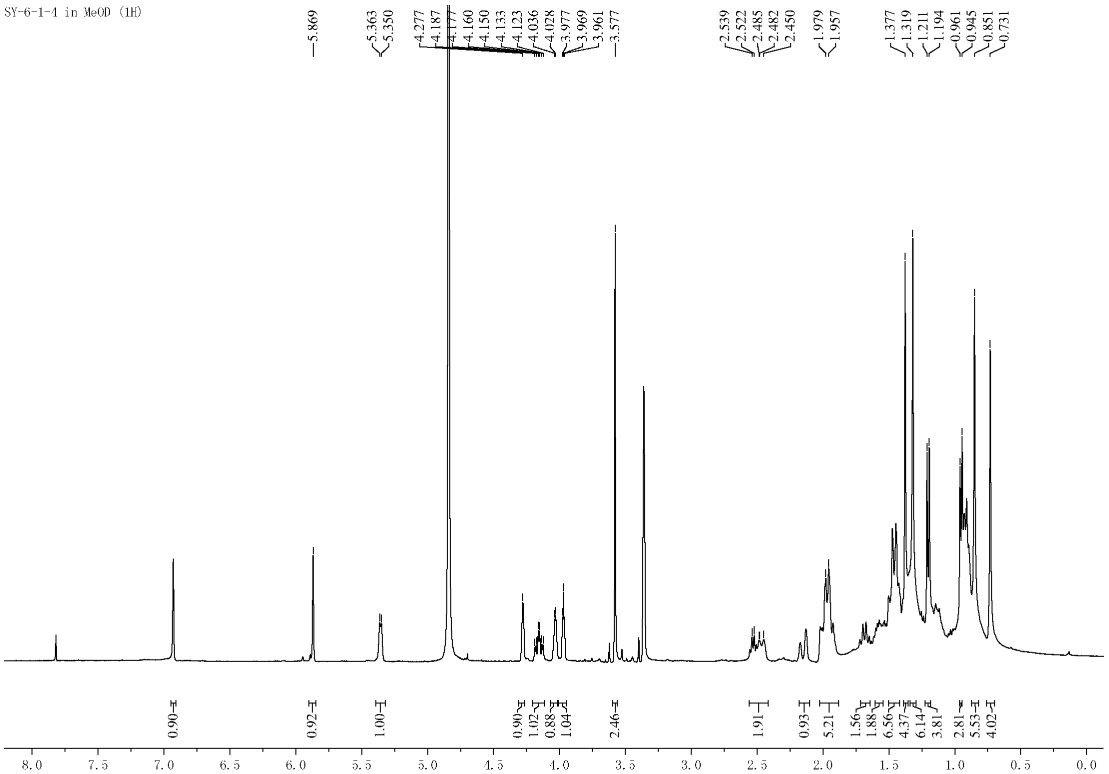

Figure S17. ^{1}H NMR (400 MHz, CD_3OD) spectrum of compound **3**.

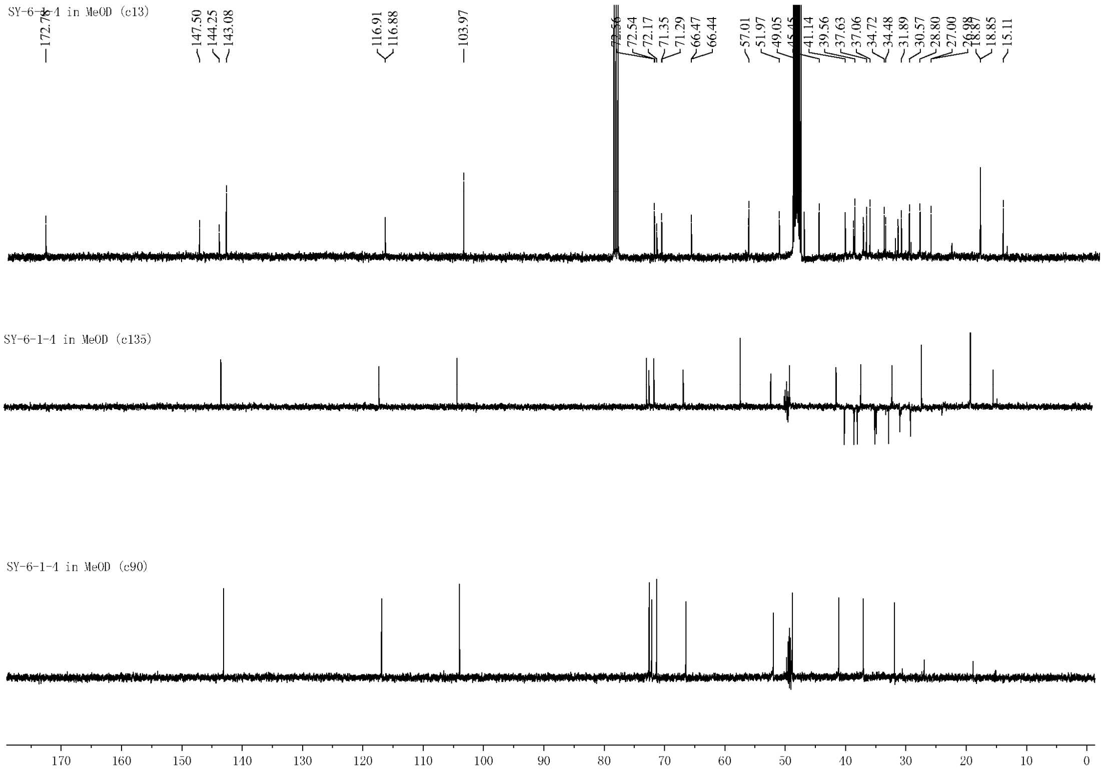

Figure S18. ^{13}C NMR and DEPT (100 MHz, CD_3OD) spectra of compound **3**.

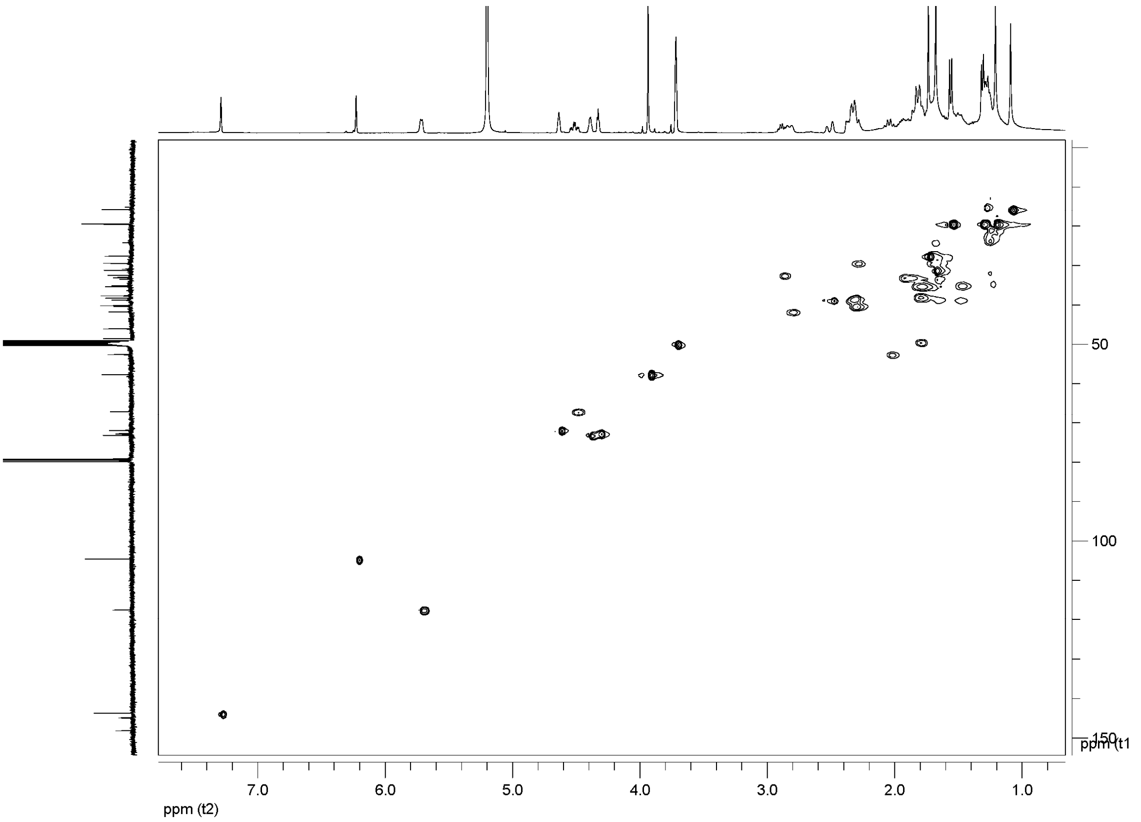

Figure S19. HSQC (CD3OD) spectrum of compound **3**.

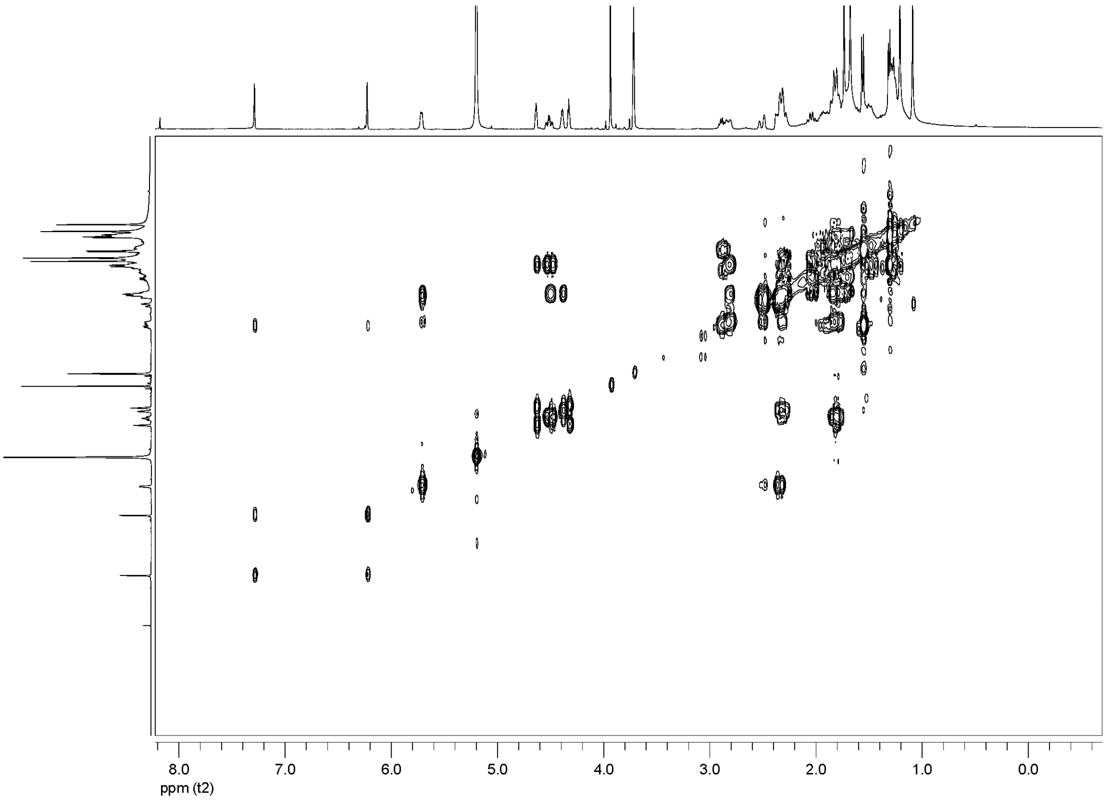

Figure S20. ^{1}H–^{1}H COSY (CD3OD) spectrum of compound **3**.

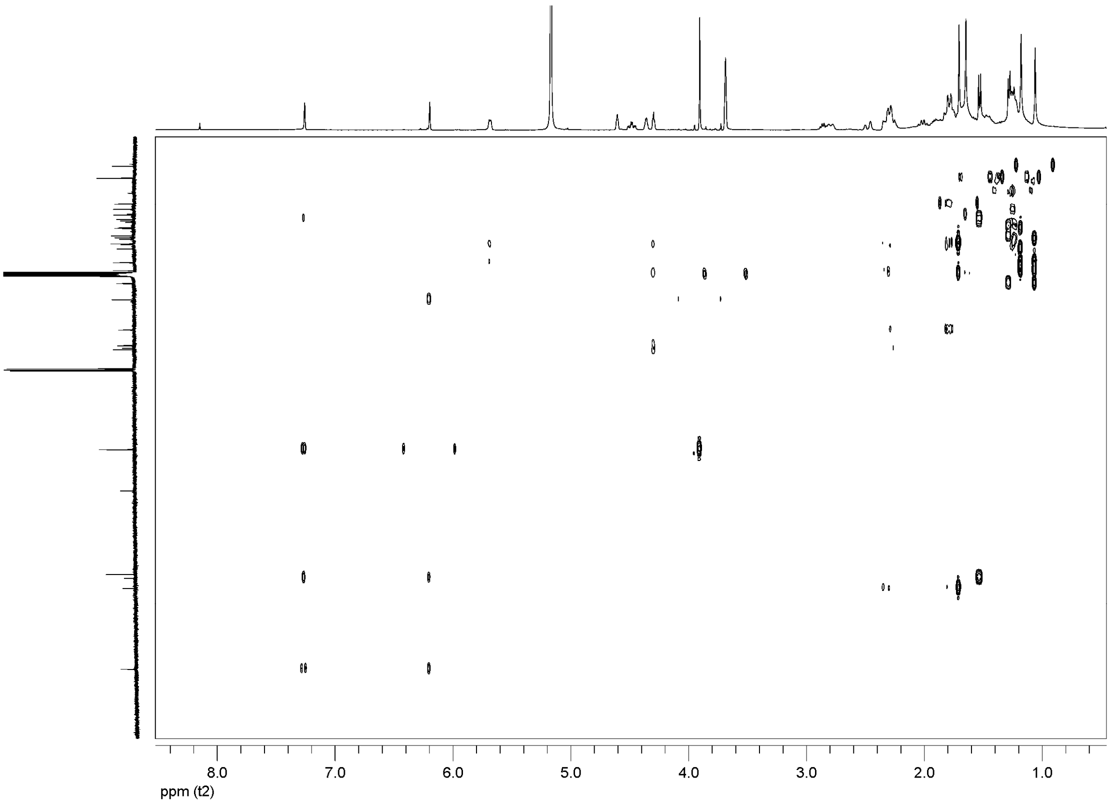

Figure S21. HMBC (CD_3OD) spectrum of compound **3**.

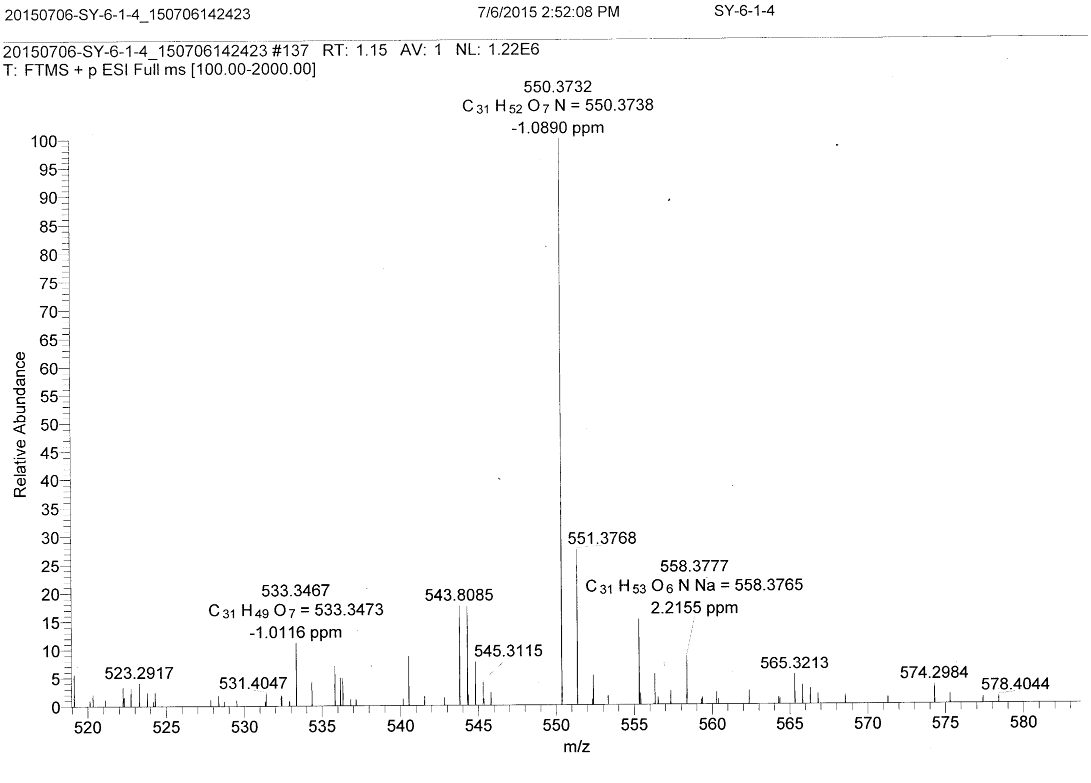

Figure S22. HRESIMS spectrum of compound **3**.
